# Supplementary material for: NF-κB Decoy ODN-Loaded Poly(Lactic-co-glycolic Acid) Nanospheres Inhibit Alveolar Ridge Resorption
Source: Int J Mol Sci. 2023 Feb 12;24(4):3699. doi: 10.3390/ijms24043699 (PMC9962103; doi:10.3390/ijms24043699)
Supplement: Supplementary file 1 [file ijms-24-03699-s001.zip › ijms-2206477-supplementary.pdf]

NF-κB Decoy ODN-Loaded Poly(Lactic-co-glycolic Acid) Nanospheres Inhibit Alveolar Ridge Resorption

Albert chun-shuo Huang, Yuji Ishida, Kai Li, Duantawan Rintanalert, Kasumi Hatano-Sato, Shuji Oishi, Jun Hosomichi, Risa Usumi-fujita, Hiroyuki Yamaguchi, Hiroyuki Tsujimoto, Aiko Sasai, Ayaka Ochi, Hajime Watanabe, Takashi Ono

Supplementary files

Supplementary Table S1. Vertical bone height loss in the extraction socket on day 7 and day 28

Supplementary Table S2. Trabecular bone analysis of extraction socket on day 7 and day 28

Supplementary Table S3. TRAP-positive cell ratio of extraction socket on day 7 and day 28

Supplementary Table S4. ALP-positive stained area ratio of extraction socket on day 7 and day 28

Supplementary Table S5. Percentage of immunopositive stained areas (%) of extraction socket on day 7 and day 28

Supplementary Table S6. Relative gene expression level of extraction socket on day 7 and day 28

Supplementary Figure S1. Schematic figure of decoy ODN and experimental timeline.

Supplementary Figure S2. Negative antigen (tissue) controls of anti-interleukin (IL)-1β, anti-tumor necrosis factor (TNF)-α, anti-transforming growth factor (TGF)-β1 and anti- receptor activator of nuclear factor-kappa B ligand (RANKL) in immunohistochemical staining.

Supplementary Table S1. Vertical bone height loss in the extraction socket on day 7 and day 28

Observation period	Vertical bone height loss	PBS				ScD				NfD				PLGA-ScD				PLGA-NfD				F (DFn, DFd)	*P* Values
		n	Mean	SD	SE	n	Mean	SD	SE	n	Mean	SD	SE	n	Mean	SD	SE	n	Mean	SD	SE		
D7	HBL (mm)	5	0.725	0.068	0.030	5	0.715	0.041	0.018	5	0.598	0.067	0.030	5	0.682	0.055	0.024	5	0.508	0.070	0.031	F (4, 20) = 11.170	P<0.0001****
	HML (mm)	5	0.784	0.040	0.018	5	0.766	0.058	0.026	5	0.689	0.059	0.027	5	0.719	0.066	0.030	5	0.606	0.035	0.016	F (4, 20) = 8.860	P=0.0003***
	HPL (mm)	5	0.523	0.027	0.012	5	0.525	0.048	0.021	5	0.499	0.081	0.036	5	0.500	0.071	0.032	5	0.410	0.048	0.022	F (4, 20) = 3.264	P=0.0325*
	HM (mm)	5	0.198	0.014	0.006	5	0.142	0.028	0.013	5	0.120	0.019	0.008	5	0.125	0.028	0.013	5	0.140	0.043	0.019	F (4, 20) = 5.985	P=0.0025**
	HD (mm)	5	0.654	0.026	0.011	5	0.643	0.036	0.016	5	0.612	0.038	0.017	5	0.615	0.063	0.028	5	0.552	0.054	0.024	F (4, 20) = 3.865	P=0.0175*
D28	HBL (mm)	5	0.993	0.070	0.031	5	0.961	0.047	0.021	5	0.939	0.084	0.038	5	0.918	0.044	0.020	5	0.838	0.057	0.025	F (4, 20) = 4.443	P=0.0099**
	HML (mm)	5	0.904	0.043	0.019	5	0.891	0.042	0.019	5	0.817	0.054	0.024	5	0.847	0.068	0.030	5	0.787	0.071	0.032	F (4, 20) = 3.739	P=0.0198*
	HPL (mm)	5	0.795	0.052	0.023	5	0.775	0.034	0.015	5	0.784	0.042	0.019	5	0.784	0.048	0.021	5	0.675	0.029	0.013	F (4, 20) = 6.985	P=0.0011**
	HM (mm)	5	0.542	0.038	0.017	5	0.493	0.042	0.019	5	0.513	0.032	0.014	5	0.474	0.064	0.029	5	0.390	0.036	0.016	F (4, 20) = 8.270	P=0.0004***
	HD (mm)	5	0.680	0.052	0.023	5	0.681	0.041	0.018	5	0.667	0.044	0.020	5	0.604	0.052	0.023	5	0.538	0.045	0.020	F (4, 20) = 8.828	P=0.0003***

Abbreviations: HBL, buccal height loss of extraction socket; HML, middle height loss of extraction socket; HPL, palatal height loss of extraction socket; HM, mesial height loss of extraction socket; HD, distal height loss of extraction socket.; D7, post-extraction day 7; D28, post-extraction day 28; PBS, PBS group; ScD, naked scrambled decoy group; NfD, naked NF-κB decoy group; PLGA-ScD, Scrambled decoy ODN-loaded PLGA Nanosphere group; PLGA-NfD, NF-κB decoy ODN-loaded PLGA nanosphere group.

Supplementary Table S2. Trabecular bone analysis of extraction socket on day 7 and day 28

Observation period	Trabecular bone Parameters	PBS				ScD				NfD				PLGA-ScD				PLGA-NfD				F (DFn, DFd)	*P* Values
		n	Mean	SD	SE	n	Mean	SD	SE	n	Mean	SD	SE	n	Mean	SD	SE	n	Mean	SD	SE		
	BV/TV (%)	5	42.11	5.06	2.26	5	41.14	3.13	1.40	5	50.54	3.17	1.42	5	43.79	4.23	1.89	5	56.77	5.65	2.53	F (4, 20) = 11.661	P<0.0001****
	BS/BV (1/mm)	5	45.31	6.31	2.82	5	48.31	6.26	2.80	5	38.16	4.59	2.05	5	51.47	5.99	2.68	5	32.65	6.17	2.76	F (4, 20) = 8.467	P=0.0004***
	BMD (mg/cm^3)	5	417.61	65.98	29.51	5	409.50	50.76	22.70	5	516.40	65.84	29.44	5	419.20	41.14	18.40	5	611.40	56.07	25.08	F (4, 20) = 12.040	P<0.0001****
	Tb.Th (μm)	5	38.91	5.26	2.35	5	38.95	6.92	3.09	5	48.02	5.23	2.34	5	42.42	4.39	1.96	5	52.52	5.59	2.50	F (4, 20) = 5.814	P=0.0028**
D7	Tb.N (1/mm)	5	9.48	2.72	1.22	5	10.20	2.46	1.10	5	14.76	3.23	1.45	5	10.52	2.36	1.05	5	17.98	3.58	1.60	F (4, 20) = 7.907	P=0.0005***
	Tb.Sp (μm)	5	59.79	5.73	2.56	5	55.68	7.00	3.13	5	49.93	7.01	3.13	5	59.64	2.65	1.19	5	45.17	4.89	2.19	F (4, 20) = 6.286	P=0.0019**
	Tb.Spac (μm)	5	123.80	11.64	5.21	5	125.00	14.27	6.38	5	105.80	7.90	3.53	5	124.90	11.17	5.00	5	94.54	11.04	4.94	F (4, 20) = 7.516	P=0.0007***
	V*m.space (mm^3)	5	1.93E-02	1.92E-03	8.58E-04	5	1.75E-02	3.23E-03	1.45E-03	5	1.37E-02	2.17E-03	9.71E-04	5	1.73E-02	3.19E-03	1.43E-03	5	1.22E-02	4.10E-03	1.83E-03	F (4, 20) = 4.633	P=0.0082**
	V*tr (mm^3)	5	1.72E-02	7.37E-03	3.30E-03	5	1.74E-02	8.17E-03	3.66E-03	5	3.12E-02	1.07E-02	4.78E-03	5	1.63E-02	6.41E-03	2.87E-03	5	4.54E-02	1.28E-02	5.71E-03	F (4, 20) = 9.232	P=0.0002***
	BV/TV (%)	5	54.42	4.96	2.22	5	51.86	5.04	2.26	5	54.15	5.57	2.49	5	54.50	8.88	3.97	5	67.39	6.89	3.08	F (4, 20) = 4.633	P=0.0082**
	BS/BV (1/mm)	5	37.09	4.97	2.22	5	40.77	3.17	1.42	5	42.23	2.95	1.32	5	39.57	5.82	2.60	5	28.09	5.99	2.68	F (4, 20) = 6.976	P=0.0011**
	BMD (mg/cm^3)	5	560.70	92.33	41.29	5	588.70	52.65	23.55	5	590.90	67.53	30.20	5	616.30	77.82	34.80	5	745.40	68.70	30.72	F (4, 20) = 4.946	P=0.0062**
	Tb.Th (μm)	5	52.41	4.38	1.96	5	49.41	4.96	2.22	5	52.27	2.75	1.23	5	51.70	3.79	1.70	5	60.80	4.81	2.15	F (4, 20) = 5.334	P=0.0043**
D28	Tb.N (1/mm)	5	16.44	3.49	1.56	5	15.62	2.32	1.04	5	18.92	3.01	1.34	5	15.57	4.17	1.86	5	22.89	3.95	1.77	F (4, 20) = 4.064	P=0.0143*
	Tb.Sp (μm)	5	29.86	3.13	1.40	5	31.29	6.27	2.80	5	26.03	4.97	2.22	5	29.66	7.44	3.33	5	18.86	4.58	2.05	F (4, 20) = 4.194	P=0.0126*
	Tb.Spac (μm)	5	84.54	7.45	3.33	5	73.02	13.13	5.87	5	68.68	10.83	4.85	5	83.79	12.84	5.74	5	53.34	13.62	6.09	F (4, 20) = 5.878	P=0.0027**
	V*m.space (mm^3)	5	1.28E-02	1.54E-03	6.88E-04	5	1.32E-02	2.40E-03	1.07E-03	5	1.15E-02	1.59E-03	7.11E-04	5	1.20E-02	1.62E-03	7.22E-04	5	7.29E-03	1.63E-03	7.31E-04	F (4, 20) = 8.845	P=0.0003***
	V*tr (mm^3)	5	4.96E-02	1.11E-02	4.95E-03	5	6.29E-02	1.09E-02	4.87E-03	5	6.27E-02	1.40E-02	6.25E-03	5	4.83E-02	1.62E-02	7.25E-03	5	7.64E-02	2.01E-02	8.98E-03	F (4, 20) = 3.001	P=0.0432*

Abbreviations: BV/TV, bone volume fraction (%); BS/BV, bone surface ratio (per mm); BMD, bone mineral density (mg/cm^3); Tb.Th, trabecular thickness (μm); Tb.N, trabecular number (per mm); Tb.Sp, trabecular separation (μm); Tb.Spac, trabecular spacing (μm); V*m.space, bone marrow space star volume (mm^3); V*tr, trabecular star volume (mm^3); D7, post-extraction day 7; D28, post-extraction day 28; PBS, PBS group; ScD, naked scrambled decoy group; NfD, naked NF-κB decoy group; PLGA-ScD, Scrambled decoy ODN-loaded PLGA Nanosphere group; PLGA-NfD, NF-κB decoy ODN-loaded PLGA nanosphere group.

Supplementary Table S3. TRAP positive cell ratio of extraction socket on day 7 and day 28

Observation period	TRAP-positive cell ratio	PBS				ScD				NfD				PLGA-ScD				PLGA-NfD				F (DFn, DFd)	*P* Values
		n	Mean	SD	SE	n	Mean	SD	SE	n	Mean	SD	SE	n	Mean	SD	SE	n	Mean	SD	SE		
D7	Number of TRAP-positive stained cells / mm2	5	25.06	3.13	1.40	5	24.08	2.95	1.32	5	17.69	3.14	1.41	5	24.53	4.85	2.17	5	10.94	2.32	1.04	F (4, 20) = 16.281	P<0.0001****
D28	Number of TRAP-positive stained cells / mm2	5	19.92	4.65	2.08	5	17.12	2.14	0.96	5	16.74	3.63	1.62	5	19.15	2.71	1.21	5	9.99	2.62	1.17	F (4, 20) = 7.169	P=0.0009***

Abbreviations: D7, post-extraction day 7; D28, post-extraction day 28; PBS, PBS group; ScD, naked scrambled decoy group; NfD, naked NF-κB decoy group; PLGA-ScD, Scrambled decoy ODN-loaded PLGA Nanosphere group; PLGA-NfD, NF-κB decoy ODN-loaded PLGA nanosphere group.

Supplementary Table S4. ALP positive stained area ratio of extraction socket on day 7 and day 28

Observation period	ALP-positive area ratio	PBS				ScD				NfD				PLGA-ScD				PLGA-NfD				F (DFn, DFd)	*P* Values
		n	Mean	SD	SE	n	Mean	SD	SE	n	Mean	SD	SE	n	Mean	SD	SE	n	Mean	SD	SE		
D7	ALP-positive stained area ratio (%)	5	7.80	4.63	2.07	5	7.63	4.53	2.03	5	9.36	3.27	1.46	5	11.94	4.33	1.93	5	20.17	3.22	1.44	F (4, 20) = 8.302	P=0.0004***
D28	ALP-positive stained area ratio (%)	5	9.85	4.30	1.92	5	8.72	3.36	1.50	5	7.32	2.59	1.16	5	8.14	3.70	1.66	5	14.58	3.02	1.35	F (4, 20) = 3.468	P=0.0262*

Abbreviations: D7, post-extraction day 7; D28, post-extraction day 28; PBS, PBS group; ScD, naked scrambled decoy group; NfD, naked NF-κB decoy group; PLGA-ScD, Scrambled decoy ODN-loaded PLGA Nanosphere group; PLGA-NfD, NF-κB decoy ODN-loaded PLGA nanosphere group.

Supplementary Table S5. Percentage of immunopositive stained areas (%) of extraction socket on day 7 and day 28

Immunohistochemical markers	Observation period	PBS				ScD				NfD				PLGA-ScD				PLGA-NfD				F (DFn, DFd)	*P* Values
		n	Mean	SD	SE	n	Mean	SD	SE	n	Mean	SD	SE	n	Mean	SD	SE	n	Mean	SD	SE		
IL-1 beta	*D7*	5	20.56	2.82	1.26	5	19.76	4.70	2.10	5	13.58	3.03	1.36	5	18.12	2.95	1.32	5	9.29	2.65	1.18	F (4, 20) = 10.240	P=0.0001***
	D28	5	8.36	2.21	0.99	5	9.03	3.74	1.67	5	6.81	1.51	0.68	5	7.81	2.33	1.04	5	2.81	1.07	0.48	F (4, 20) = 5.458	P=0.0039**
TNF-alpha	*D7*	5	15.54	2.97	1.33	5	14.28	2.34	1.05	5	11.71	2.33	1.04	5	13.43	2.23	0.99	5	6.44	1.91	0.85	F (4, 20) = 11.101	P<0.0001****
	D28	5	10.86	1.66	0.74	5	10.51	2.30	1.03	5	9.61	2.06	0.92	5	9.99	3.00	1.34	5	4.28	1.67	0.75	F (4, 20) = 7.602	P=0.0007***
TGF-beta 1	*D7*	5	18.30	3.03	1.36	5	17.15	2.71	1.21	5	22.91	4.11	1.84	5	17.92	3.59	1.60	5	30.62	3.67	1.64	F (4, 20) = 13.270	P<0.0001****
	D28	5	21.36	3.47	1.55	5	22.11	4.29	1.92	5	23.75	2.04	0.91	5	23.23	3.44	1.54	5	31.75	3.88	1.74	F (4, 20) = 7.139	P=0.0010***
RANKL	*D7*	5	16.34	2.39	1.07	5	18.63	2.06	0.92	5	13.82	2.00	0.89	5	16.90	2.19	0.98	5	9.77	1.80	0.80	F (4, 20) = 13.450	P<0.0001****
	D28	5	12.27	1.67	0.75	5	11.99	2.04	0.91	5	12.21	2.61	1.17	5	13.43	1.48	0.66	5	8.43	2.07	0.93	F (4, 20) = 4.444	P=0.0099**

Abbreviations: D7, post-extraction day 7; D28, post-extraction day 28; PBS, PBS group; ScD, naked scrambled decoy group; NfD, naked NF-κB decoy group; PLGA-ScD, Scrambled decoy ODN-loaded PLGA Nanosphere group; PLGA-NfD, NF-κB decoy ODN-loaded PLGA nanosphere group.

Supplementary Table S6. Relative gene expression level of extraction socket on day 7 and day 28

RT-qPCR markers	Observation period	PBS				ScD				NfD				PLGA-ScD				PLGA-NfD				Kruskal-Wallis statistic	*P* Values
		n	Mean	SD	SE	n	Mean	SD	SE	n	Mean	SD	SE	n	Mean	SD	SE	n	Mean	SD	SE		
IL-1 beta	*D7*	5	1.04	0.22	0.10	5	1.09	0.37	0.16	5	0.81	0.15	0.07	5	0.95	0.20	0.09	5	0.52	0.21	0.10	12.68	P=0.0130*
	D28	5	1.05	0.21	0.10	5	0.92	0.24	0.11	5	0.86	0.16	0.07	5	0.90	0.15	0.07	5	0.59	0.19	0.09	11.44	P=0.0221*
TNF-alpha	*D7*	5	1.07	0.40	0.18	5	1.16	0.27	0.12	5	0.89	0.38	0.17	5	1.29	0.35	0.16	5	0.46	0.24	0.11	12.90	P=0.0118*
	D28	5	0.96	0.16	0.07	5	1.06	0.27	0.12	5	1.03	0.43	0.19	5	1.05	0.32	0.14	5	0.50	0.12	0.05	11.87	P=0.0183*
TGF-beta 1	*D7*	5	1.10	0.25	0.11	5	1.04	0.19	0.09	5	1.36	0.22	0.10	5	1.17	0.19	0.09	5	1.53	0.22	0.10	9.70	P=0.0459*
	D28	5	1.03	0.23	0.10	5	1.17	0.18	0.08	5	1.20	0.15	0.07	5	1.32	0.26	0.12	5	1.68	0.39	0.18	10.28	P=0.0360*
RANKL	*D7*	5	0.99	0.30	0.13	5	1.06	0.27	0.12	5	0.69	0.17	0.07	5	1.01	0.24	0.11	5	0.49	0.20	0.09	12.55	P=0.0137*
	D28	5	1.11	0.18	0.08	5	1.05	0.19	0.09	5	0.97	0.18	0.08	5	1.01	0.22	0.10	5	0.69	0.13	0.06	10.70	P=0.0301*
OPG	*D7*	5	1.02	0.20	0.09	5	0.97	0.12	0.05	5	0.86	0.17	0.08	5	0.93	0.25	0.11	5	0.62	0.13	0.06	10.27	P=0.0361*
	D28	5	1.01	0.14	0.06	5	1.08	0.10	0.04	5	0.99	0.13	0.06	5	0.96	0.21	0.09	5	0.73	0.08	0.03	10.55	P=0.0322*
RANKL/OPG ratio	*D7*	5	1.07	0.34	0.15	5	1.09	0.27	0.12	5	0.83	0.27	0.12	5	1.20	0.35	0.16	5	0.62	0.19	0.09	10.51	P=0.0327*
	D28	5	1.12	0.16	0.07	5	0.88	0.23	0.10	5	0.89	0.19	0.09	5	1.04	0.28	0.12	5	0.66	0.17	0.08	10.63	P=0.0311*

Abbreviations: D7, post-extraction day 7; D28, post-extraction day 28; PBS, PBS group; ScD, naked scrambled decoy group; NfD, naked NF-κB decoy group; PLGA-ScD, Scrambled decoy ODN-loaded PLGA Nanosphere group; PLGA-NfD, NF-κB decoy ODN-loaded PLGA nanosphere group.

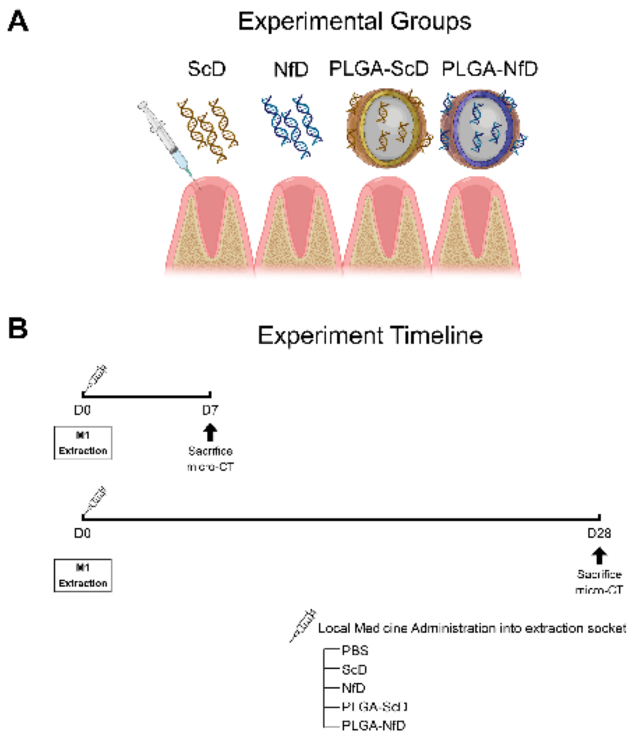

Supplementary Figure S1.

A, Schematic figure of decoy ODN nuclear medicine administered in experimental groups.

B, Experimental timeline.

Abbreviations: D0, immediate after extraction; D7, post-extraction day 7; D28, post-extraction day 28; PBS, PBS group; ScD, naked scrambled decoy group; NfD, naked NF-κB decoy group; PLGA-ScD, Scrambled decoy ODN-loaded PLGA Nanosphere group; PLGA-NfD, NF-κB decoy ODN-loaded PLGA nanosphere group.

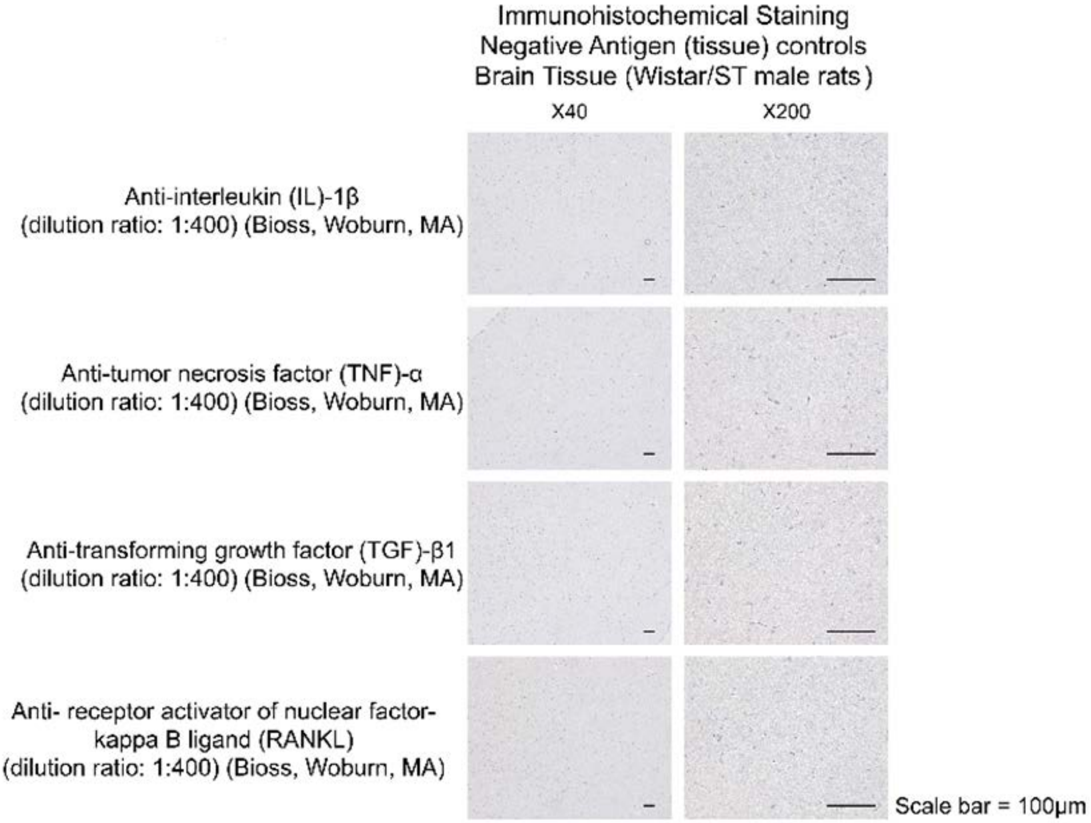

Supplementary Figure S2.

Negative antigen (tissue) controls of anti-interleukin (IL)-1β (dilution ratio: 1:400) (Bioss, Woburn, MA), anti-tumor necrosis factor (TNF)-α (dilution ratio: 1:400) (Bioss, Woburn, MA), anti-transforming growth factor (TGF)-β1 (dilution ratio: 1:400) (Bioss, Woburn, MA) and anti- receptor activator of nuclear factor-kappa B ligand (RANKL) (dilution ratio: 1:400) (Bioss, Woburn, MA) in immunohistochemical staining. Brain tissues of Wistar/ST male rats were shown in the figure. Original magnification 40x and 200x. Scale Bar = 100 μm.
